# Supplementary material for: SSL5-AnxA5 Fusion Protein Constructed Based on Human Atherosclerotic Plaque scRNA-Seq Data Preventing the Binding of Apoptotic Endothelial Cells, Platelets, and Inflammatory Cells
Source: Biomedicines. 2024 Dec 24;13(1):8. doi: 10.3390/biomedicines13010008 (PMC11763200; doi:10.3390/biomedicines13010008)
Supplement: Supplementary file 1 [file biomedicines-13-00008-s001.zip › biomedicines-3365320-supplementary.pdf]

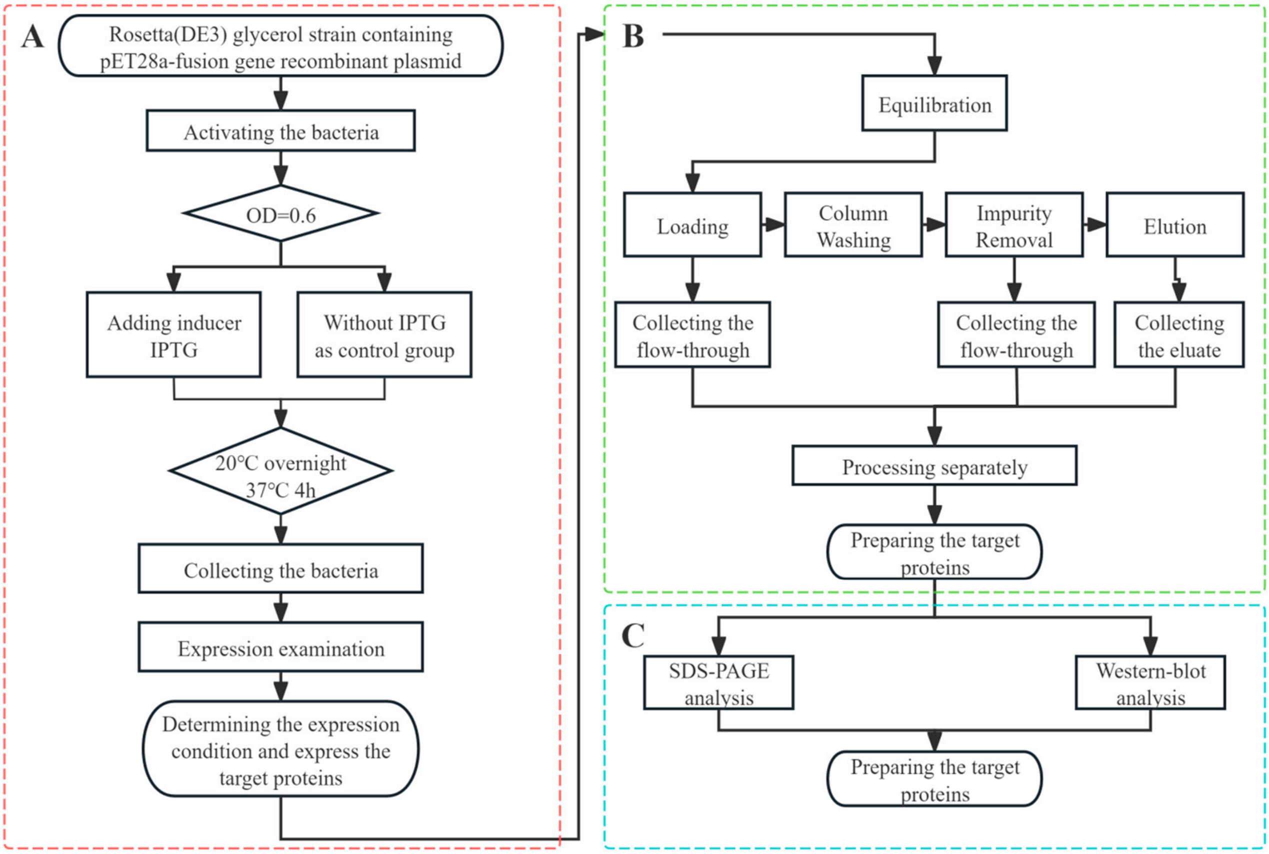

Supplementary Figure S1. The process of SSL5-AnxA5 preparation.

A, The process of SSL5-AnxA5 expression. **B**, The process of SSL5-AnxA5 purification. **C**, The process of SSL5-AnxA5 verification.

Abbreviation: IPTG, isopropyl-β-D-thiogalactoside, a strong and stable exogenous gene expression inducer.

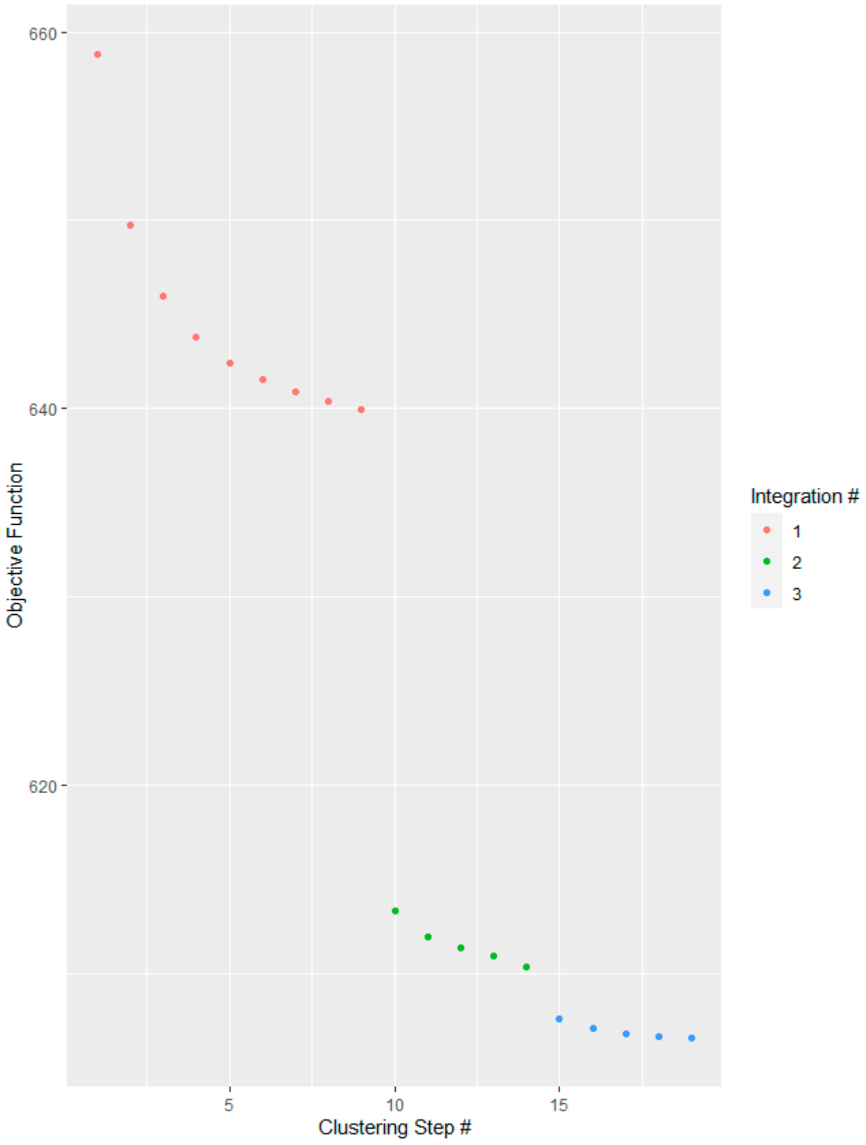

Supplementary Figure S2. Objective function convergence during batch effect correction using the Harmony algorithm.

The plot shows the decrease in the objective function value over the clustering steps for three different integration rounds (denoted by different colors: red for round 1, green for round 2, and blue for round 3). As the clustering progresses, the objective function converges, indicating successful integration and batch effect correction across datasets. This demonstrates that Harmony effectively minimizes batch effects, resulting in comparable cell type distributions between the ACS and SAP groups.

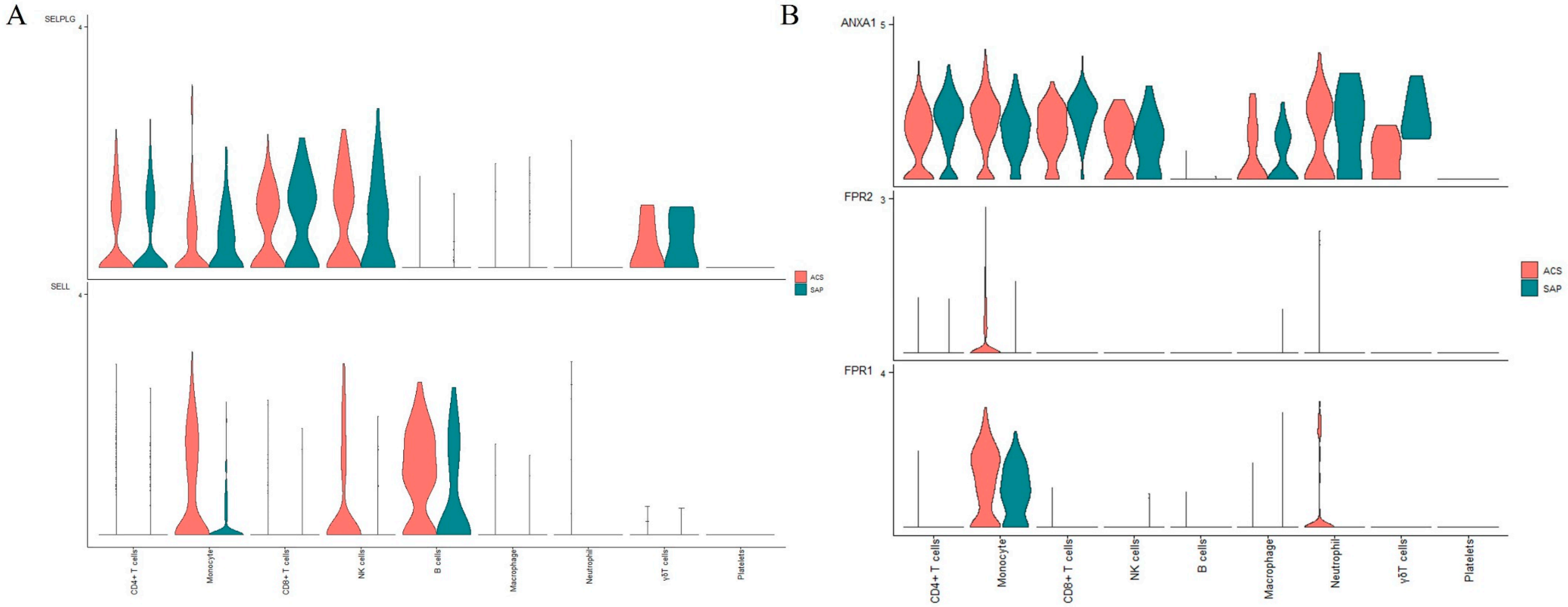

Supplementary Figure S3. Violin plots comparing the expression levels of key genes involved in the SELPLG and ANNEXIN signaling pathways across various cell types in ACS and SAP groups.

A, Violin plots displaying the expression of SELPLG and SELL in different cell types. The expression of SELPLG is higher in monocytes and NK cells in the ACS group compared to SAP, while SELL shows higher expression in monocytes, B cells and NK cells in ACS. **B,** Violin plots showing the expression of ANXA1, FPR1, and FPR2, critical components of the ANNEXIN signaling pathway. ANXA1 is broadly expressed in both ACS and SAP groups across multiple cell types, including CD4+ T cells, monocytes, and macrophages, with slightly elevated expression in ACS. FPR1 is primarily expressed in monocytes and neutrophils, while FPR2 shows increased expression in monocytes and neutrophils, both with higher levels observed in ACS compared to SAP.

Supplementary Table S1. The specific composition of the buffers in process of SSL5-AnxA5 purification.

**Buffer**	**Composition**
Binding buffer	8 M Urea, 50 mM Tris, 300 mM NaCl, pH 8.0
Washing buffer	8 M Urea, 50 mM Tris, 300 mM NaCl, 20/50 mM Imidazole, pH 8.0
Elution buffer	8 M Urea, 50 mM Tris, 300 mM NaCl, 500 mM Imidazole, pH 8.0
